# Circulating Cell-Free DNA-Based Detection of Tumor Suppressor Gene Copy Number Loss and Its Clinical Implication in Metastatic Prostate Cancer

**DOI:** 10.3389/fonc.2021.720727

**Published:** 2021-08-24

**Authors:** Xiaoxi Dong, Tiantian Zheng, Minhua Zhang, Chao Dai, Lili Wang, Lei Wang, Ruipeng Zhang, Yuan Long, Danyi Wen, Feng Xie, Yue Zhang, Yong Huang, Jianguo Dong, Huan Liu, Pan Du, Bonnie L. King, Winston Tan, Shidong Jia, Chris X. Lu, Manish Kohli, Haitao Wang, Jianjun Yu

**Affiliations:** ^1^Predicine Inc., Hayward, CA, United States; ^2^Department of Translational Medicine, Laekna Therapeutics Shanghai Co., Ltd., Shanghai, China; ^3^Department of Oncology, Second Hospital of Tianjin Medical University, Tianjin, China; ^4^Department of New Assay Development, Shanghai Lide Biotech Co., Ltd., Shanghai, China; ^5^Shanghai Lide Biotech Co., Ltd., Shanghai, China; ^6^Huidu Shanghai Medical Sciences Ltd., Shanghai, China; ^7^Department of Biology, Shanghai Lide Biotech Co., Ltd., Shanghai, China; ^8^Division of Medical Oncology, Department of Medicine, Mayo Clinic, Jacksonville, FL, United States; ^9^Laekna Therapeutics Shanghai Co., Ltd., Shanghai, China; ^10^Division of Oncology, Department of Internal Medicine, Huntsman Cancer Institute, University of Utah, Salt Lake City, UT, United States

**Keywords:** liquid biopsy, circulating tumor DNA, copy number loss, prostate cancer, NGS

## Abstract

Current liquid biopsy assays lack sufficient sensitivity to detect copy number loss, which limits the interrogation of critical tumor suppressor gene deletions during cancer progression and treatment. Here we describe a liquid biopsy assay with improved sensitivity for detection of copy number loss in blood samples with low levels of circulating tumor DNA, and demonstrate its utility by profiling *PTEN*, *RB1*, and *TP53* genetic loss in metastatic prostate cancer patients.

## Introduction

The evaluation of *PTEN*, *RB1*, and *TP53* copy number loss in primary tumor tissue samples during the progression of prostate cancer has established the clinical utility of these tumor suppressor genes as biomarkers for prognosis, response to therapy, and therapeutic resistance (1, 2). Due to the challenges of obtaining high-purity tumor tissue from skeletal metastatic sites, which are common in prostate cancer (3), liquid biopsy constitutes a surrogate approach for tracking genomic alterations in tumor cells during treatment and metastatic progression (4). However, cell-free DNA (cfDNA) derived from tumor cells is often present at very low quantities in liquid biopsy samples, making it challenging to detect copy number loss (5). Thus, the development of a sensitive liquid biopsy test for the detection of tumor suppresser gene loss represents a critical unmet need.

We have developed a next-generation sequencing (NGS)-based cfDNA assay (PredicineCARE) that detects copy number loss with high sensitivity in the blood. Here we describe the evaluation of our assay in two independent metastatic castrate-resistant prostate cancer (mCRPC) patient cohorts (**Figure S1**) to (i) compare detection of copy number loss events in 15 matched pairs of tumor tissue and plasma cfDNA samples with orthogonal low-pass whole genome sequencing (LP-WGS); (ii) further assess the concordance of the assay and LP-WGS with immunohistochemical detection of PTEN protein loss in tumor tissues; and (iii) evaluate the use of the assay to detect *PTEN*, *RB1*, and *TP53* copy loss events in liquid biopsy samples collected from a second cohort of 52 mCRPC patients in association with patient outcomes. The sensitive detection of copy number loss in liquid biopsy samples is critical to the management of mCRPC as together these three alterations are seen in up to 34% of patients and are associated with poor prognosis (4). The development of an accurate assay constitutes an important step towards the practice of precision medicine in the mCRPC state.

## Methods

### Orthogonal Assay Validation Study

Concordance of genomic aberrations between plasma and tissue samples was assessed in a prospective cohort of 15 ARPI-resistant metastatic prostate cancer patients at the Second Hospital of Tianjin Medical University, China (NCT03786848). Each patient underwent imaging-guided core needle biopsy sampling from an accessible metastatic lesion. Paired collection of peripheral blood (10 ml in an EDTA-containing tube) was obtained on the day of tissue biopsy. Metastatic tissue and blood samples underwent both targeted panel sequencing and low-pass whole genome sequencing (LP-WGS); tissue samples underwent additional PTEN immunohistochemistry (IHC) assessment. Details of tissue sample processing and IHC assessment are described in **Supplementary Methods**.

### Clinical Utility Study

Plasma samples were collected from 52 prostate cancer patients prospectively enrolled at the Mayo Clinic, Rochester, Minnesota, between September 2009 and March 2014, with metastatic prostate cancer, which included the sub-cohort of mCRPC patients with either biochemically or radiographically progressive disease after failure of androgen-deprivation therapy. This target mCRPC sub-cohort was enrolled prior to the commencement of chemotherapy for the mCRPC state, and samples were processed uniformly as has been previously reported (4). Study approval was obtained from the Mayo Clinic Institutional Review Board (MC IRB # 09-1989 00; Title: “*Study of Molecular Circulatory Biomarkers in Hormone Sensitive and Castration Recurrent Prostate Cancer*”), and all participants provided a written informed consent prior to sample collection. Baseline clinical characteristics for all patients are presented in **Table S2**. Median follow-up time for non-deceased patients was 80.7 months.

### Plasma-Based cfDNA Molecular Profiling

All plasma samples obtained for NGS profiling were collected with the approval of Institutional Review Boards (IRB). ctDNA sequencing from plasma was performed using the harmonized CLIA-certified PredicineCare™ NGS assay in laboratories in the US and China, respectively. The data analysis of US and China patients was conducted in the US and China, respectively. Fisher’s exact test was performed to compare mutational frequencies across populations. Relevant to this study, the assay targeted all coding regions of *PTEN, RB1*, and *TP53* (see **Supplementary Table 3** for full gene list). Simultaneous sequencing of matched white blood cells was also undertaken. A detailed description of DNA extraction, sequencing, and bioinformatics analysis, including description of the methods used to optimize copy number calls and calculation of ctDNA fraction, is presented in the **Supplementary Methods**. Orthogonal sequencing was performed using LP-WGS, as also described in **Supplementary Methods**.

### Tissue-Based Genomic DNA Molecular Profiling

Tumor cell percentage was evaluated for all 15 tissue FFPE samples. DNA was isolated from FFPE blocks using the QIAamp DNA FFPE tissue kit (Qiagen). Following quantification (Qubit 2.0 fluorometer) and quality assessment (Bioanalyzer 2100), 500 ng of isolated DNA underwent shearing to ~300 bp fragments using the Bioruptor Pico sonication device (Diagenode, Denville, NJ, USA). Post-sonication quality control was performed (Bioanalyzer 2100), and 200 ng FFPE DNA was utilized for library preparation. Targeted capture, sequencing, and bioinformatics analysis of tumor tissue were as the cfDNA workflow outlined above.

### Clinical Outcomes and Statistical Analysis

Kaplan-Meier survival estimates and multivariable Cox proportional-hazards models were used to assess the association between *PTEN*, *RB1*, and *TP53* aberrations and overall survival (OS; time from sample collection date until death from any cause). Where an event had not occurred at the time of data analysis, survival outcomes were right-censored at the date of last patient contact. Statistical significance was determined as *P* < 0.05 by log-rank test. All analyses were performed in R v4.0.1.

## Results

### Concordance of Copy Number Variation (CNV) Between cfDNA Plasma and Tissue Biopsy by NGS

The PredicineCARE test is a hybrid-capture-based NGS-targeted liquid biopsy assay in addition to its standard application to FFPE tissue. We have previously validated the assay for detecting single nucleotide variation (SNV) and CNV using standard reference materials and demonstrated high assay sensitivity and positive predictive value (PPV) (6). In brief, the assay is able to detect single nucleotide variation (SNV) including short insertion and deletions (Indels) at an allelic frequency (AF) of 0.25%, copy number gain at 2.23 (equivalent to six copies for a given gene at 5% tumor fraction [TF]), and copy number loss at 1.75 (equivalent to homozygous deletion of a gene at approximately 10–12.5% TF) with both sensitivity and PPV >95% (**Table S1**).

**Figure 1:**
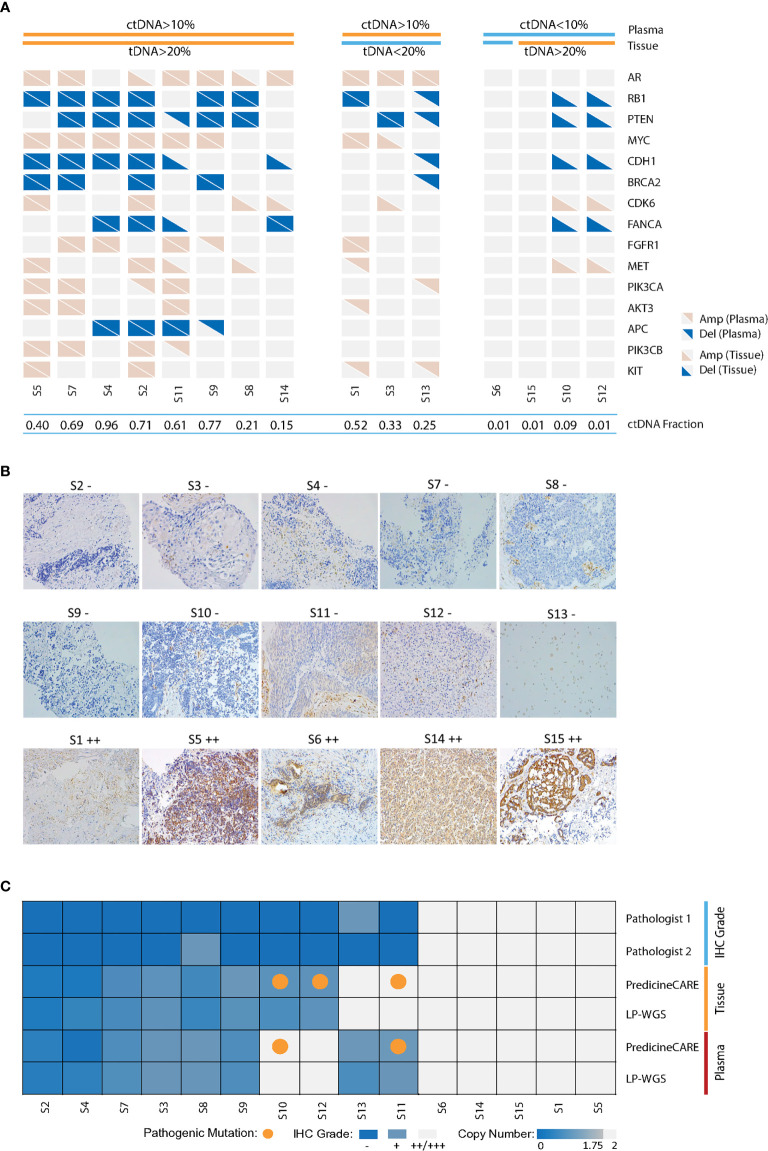
Validation of cfDNA-based gene copy number variation. Paired tumor tissue-plasma samples collected from 15 metastatic castration-resistant prostate cancer patients were analyzed by PredicineCARE, LP-WGS, and IHC assays. **(A)** Landscape of gene copy number variations, including amplification and deletion events detected by the PredicineCARE assay in tissue and plasma samples. Genes altered in >10% of the samples are shown. Samples are grouped according to circulating tumor DNA (ctDNA) and tissue tumor DNA (tDNA) fractions. ctDNA fractions were estimated by LP-WGS and mutation allele frequency reported by the PredicineCARE assay. Tissue tumor cell percentages (tDNA) were estimated by pathological reviews. Amp, gene amplification (copy number gain); Del, gene deletion (copy number loss). **(B)** *PTEN* expression in 15 prostate cancer tissues as detected by immunohistochemistry (×20). Pathology score was defined as the product of a staining intensity score (0–3) and stained area score (0–3). If the score was ≤1, the sample was characterized as negative (−). If the score was >1, the result was positive (Score 1–3, grade +; Score 4–6, grade ++; Score 7–9, grade +++). Two pathologists reviewed the slides independently, and the average score was considered as the final score for a given case. **(C)** Agreement of *PTEN* loss between tissue and blood-based detection for the 15 pairs of samples. “IHC grade” shows *PTEN* protein expression by immunohistochemical staining. *PTEN* gene copy loss at the DNA level was evaluated in tissue and plasma samples using the PredicineCARE assay and low-pass whole-genome sequencing assay (LP-WGS).

In this study, we addressed the concordance of CNV in paired tissue biopsy and blood samples as detected by PredicineCARE. We further evaluated the correlation between copy number loss detected by NGS and protein loss reported by immunohistochemistry (IHC) analysis. Paired tumor biopsy and plasma sample sets were collected from 15 mCRPC patients during their clinical visits (orthogonal validation cohort, Methods, and **Table S2**). Among the genes covered by the panel (**Table S3**), copy number gain and loss were successfully identified in the plasma samples and tumor tissue samples, including gain of androgen receptor (*AR*, n = 8, 53%) and *MYC* (n = 7, 46%) and loss of key tumor suppressor genes such as *RB1* (n = 8, 53%), *PTEN* (n = 8, 53%), and *APC* (n = 4, 27%), as shown in **Figure 1A**.

The assessment of concordance of genomic alterations between tissue and plasma is heavily influenced by the tumor fraction (TF) in each specimen and tissue type. When TF decreases disproportionately in the tissue biopsy or plasma samples, the concordance declines. For example, plasma samples from patients S10 and S12 contained very low circulating tumor DNA (ctDNA) fraction. No CNV was detected in these plasma cfDNAs. In contrast, CNVs were detected in the paired tissue biopsy samples (with TF >20%) from patients S10 and S12. Similarly, tumor tissue (<20% TF, **Figure 1A**) from patient S13 harbored few CNV events, while CNVs were detected in matched plasma (>10% ctDNA, **Figure 1A**). These findings were also confirmed by orthogonal low-pass whole genome sequencing (LP-WGS) of both plasma cfDNA and tissue DNA (tDNA), as illustrated in **Figure S2**. As such, concordance between matched sets declined when either compartment exhibited a disproportionately low TF. However, strong agreement was observed for the samples with high tumor fractions in both compartments (cfDNA-TF >10% and tDNA-TF >20%), with nearly 100% concordance for patients S2, S4, and S7 (**Figure 1A**), in keeping with high correlations of estimated copy numbers in these samples (**Figure S3**). Taken together, these results demonstrated good concordance of copy number variations detected by both assays in matched tissue and plasma samples with comparable tumor fractions.

### Concordance Between PTEN Copy Number Loss and PTEN Protein Loss in the Paired Tumor Tissue Biopsy and Plasma Samples

Next, we set out to verify *PTEN* loss status by PredicineCARE and by immunohistochemistry (IHC) in the 15 samples in the above orthogonal validation cohort. PTEN IHC scoring was performed by two independent pathologists (JD and HL) (**Table S4** and **Figure 1B**). Among the 15 tumor tissue biopsy samples analyzed by IHC, 10 samples were characterized for loss of PTEN protein expression (**Figures 1B, C**). The PredicineCARE assay and LP-WGS demonstrated 100% agreement on detecting *PTEN* loss across matched tissue and plasma samples (**Figure 1C**). Notably, both assays confirmed copy number deletion of *PTEN* in 8/8 IHC PTEN-loss patients with plasma TF above 10%. Moreover, all five samples characterized as PTEN IHC-positive were confirmed as copy number neutral by NGS (**Figure 1C**). Collectively, the assay revealed an overall 86.7% concordance with IHC on detecting *PTEN* loss (80% sensitivity, 100% specificity, and 100% PPV, **Figure 1C**).

### Detection of *RB1*, *PTEN*, and *TP53* Copy Number Loss in the Blood Samples From a Metastatic Prostate Cancer Patient Cohort

**Figure 2:**
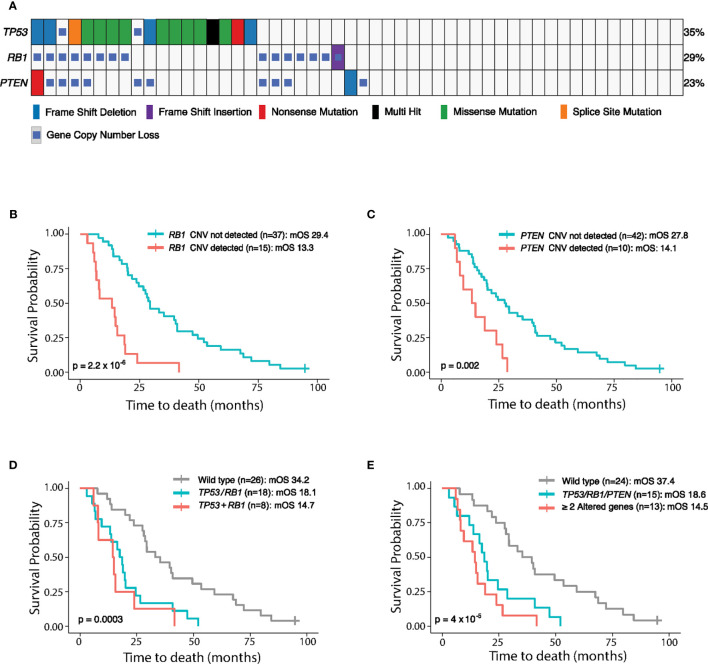
Genomic alterations of *TP53*, *RB1*, and *PTEN* detected in 52 mCRPC plasma samples collected at the Mayo Clinic and their association with patient outcome. **(A)** Genomic landscape of *PTEN*, *RB1*, and *TP53* in 52 mCRPC patients, including copy number variations (CNVs), single nucleotide variations (SNVs), and short insertions/deletions, reported by the cfDNA-based PredicineCARE assay. Blood samples were collected from patients before chemotherapy treatment. The percentage of samples having aberrations (SNV + CNV) in each gene is listed to the right of the heatmap. **(B–E)** Kaplan-Meier analysis of overall survival (OS) according to *PTEN*, *RB1*, and *TP53* loss status. OS is plotted for different patient groups classified according to **(B)** *RB1* copy loss status, **(C)** *PTEN* copy loss status, **(D)** alterations (SNV and/or CNV) in *TP53* and/or *RB1*, and **(E)** alterations (SNV and/or CNV) in one *vs*. more than one of the *PTEN*, *RB1*, and *TP53* genes. mOS, median overall survival. *TP53/RB1*: either *TP53* or *RB1* was aberrant. *TP53+RB1*: both *TP53* and *RB1* were aberrant. *TP53/RB1/PTEN*: any one of *TP53*, *RB1*, or *PTEN* was aberrant. ≥2 Altered Genes: at least two genes among the *TP53*, *RB1*, or *PTEN* triad harbored genomic aberrations.

We further analyzed 52 plasma samples from mCRPC patients collected at the Mayo Clinic on an Institutional Review Board (IRB)-approved study after obtaining written consent (4). The most common genes characterized for copy number loss were *RB1* and *PTEN*, with prevalence of 28.8 and 19.2%, respectively (**Figure 2A**). Copy number loss of *TP53* was also detected in two patients (**Figure 2A**). As loss of gene function can be introduced by gene deletion and/or mutation events, we examined both aberrations together (**Figure 2A**), which resulted in the combined prevalence rates of 35% for *TP53*, 29% for *RB1*, and 23% for *PTEN*, respectively. Eight patients (15%) carried alterations in both *TP53* and *RB1*, and five patients (9.6%) harbored alterations in all three genes (*PTEN*, *RB1*, and *TP53).* These results demonstrated the capability of our assay to detect copy loss events and capture the genomic alterations for both tumor suppressor genes and oncogenes.

### Copy Number Loss of *RB1*, *TP53*, and *PTEN* Correlates With Worse Clinical Outcome

Overall survival (OS) was then analyzed for the above 52 mCRPC patients in relation to copy loss events detected in the *RB1* and *PTEN* genes prior to the commencement of chemotherapy. Remarkably, individual gene copy number loss in either the *RB1 or PTEN* gene was strongly associated with shorter OS (p = 2.2 × 10^−6^ for *RB1* loss, p = 0.002 for *PTEN* loss, **Figures 2B, C** and **Table S6**). Importantly, this association remains significant in multivariate models after adjusting for clinical parameters including age, ctDNA fraction, cfDNA yield, and Gleason Score (**Table S6**).

We further investigated whether cumulative loss in *TP53*, *RB1*, and *PTEN* is more strongly associated with patient outcome. Notably, co-loss of both *RB1* and *TP53* led to a trend of shorter OS compared to single gene loss (median OS [mOS]: 14.7 months *vs* 18.1 months, **Figure 2D** and **Table S6**). Moreover, OS was significantly reduced in patients with loss of any one of the *PTEN*, *TP53*, and *RB1* genes relative to patients without aberrations in these genes (P = 4 × 10^−5^, mOS: 18.6 months *vs* 37.4 months, **Figure 2E** and **Table S6**), and patients with ≥2 altered genes in this triad experienced the shortest overall survival (mOS: 14.5 months, **Figure 2E**).

Functional loss of the *RB1*, *TP53*, and *PTEN* genes is associated with aggressive prostate cancer characterized by the emergence of resistance to androgen deprivation therapy and the acquisition of neuroendocrine histomorphology (7). Co-loss of *RB1* and *TP53* genes is required to induce tumor cell lineage plasticity-related anti-androgen drug resistance in prostate cancer (7). Indeed, among the 15 mCRPC patients in the orthogonal validation cohort, eight tumors exhibiting neuroendocrine-like morphology were characterized for *PTEN* copy number loss and six of them for co-loss of *RB1* and *TP53* genes (by mutations and/or copy number loss).

## Discussion

Loss of tumor suppressor genes, which comprise the largest category of cancer driver genes, plays a central role in cancer development, progression, and the emergence of drug resistance (2, 8). Liquid biopsy provides a non-invasive way to assess tumor genetic aberrations but has been limited in detecting gene copy number loss in the samples with low ctDNA content (5). Alterations in the *RB1*, *TP53*, *and PTEN* tumor suppressor gene triad have been linked with the aggressive mCRPC subtype characterized by neuroendocrine histopathology (7). Loss of these genes as biomarkers for prognosis, response to therapy, and therapeutic resistance has been previously established using tumor tissues (9, 10). However, detection of *RB1*, *TP53*, or *PTEN* copy number loss has been challenging in blood samples with ctDNA fractions below 30% (1).

Here we have described a liquid biopsy assay to detect copy number loss events in blood samples from mCRPC patients, including those with ctDNA fractions below 20%. The assay also demonstrated strong concordance with IHC in detecting *PTEN* loss. Interestingly, most tissues characterized for *PTEN* loss exhibited traits of neuroendocrine-like tumor cells. A limitation of the current study is that only 15 matched tissue/plasma samples were analyzed for concordance of *PTEN* gene copy and protein loss by our assay and IHC, respectively, due to the challenges of obtaining tissue biopsy from metastatic lesions of mCRPC patients. Thus, the concordance of these assays in detecting loss of *PTEN* warrants further validation in a larger study. To our knowledge, very few studies have demonstrated the prognostic or predictive value of gene loss detected in liquid biopsy due to the low sensitivity of the assay (11). The detection of tumor suppressor gene loss by our new assay enabled the correlation of *PTEN*1 and *RB1* loss with worse patient outcomes in a cohort of mCRPC patients, supporting its potential use in clinical development and routine practice.

In conclusion, we have introduced a cfDNA-based liquid biopsy assay for the detection of copy number loss in blood samples from mCRPC patients, with a level of sensitivity that can enable practical clinical applications, including predicting patient survival, selecting therapies for treatment, and understanding subsequent resistance. This capability makes the assay a powerful tool for improving personalized cancer care and clinical drug development.

## Data Availability Statement

The original contributions presented in the study are included in the article/**Supplementary Material**. Further inquiries can be directed to the corresponding author.

## Ethics Statement

The studies involving human participants were reviewed and approved by the Clinical And Translational Research Ethics Consultation (CLINTREC), Mayo Clinic. The patients/participants provided their written informed consent to participate in this study.

## Author Contributions

Study conception and design: XD, MZ, SJ, CL, MK, HW, and JY. Data acquisition: MK, MZ, LLW, LW, YL, YZ, and WT. Data analysis and interpretation: XD, TZ, MZ, CD, FX, BK, JD and HL. Manuscript drafting: XD. Manuscript revision and intellectual contribution: MZ, PD, MK, WT, SJ, CL, JY, and BK. Statistical analysis: XD and TZ. Obtaining funding: SJ, CL, HW, and JY. Administrative, technical, and material support: LLW, LW, RZ, and YL. Study supervision: DW, YH, WT, SJ, CL, MK, HW, and JY. All authors contributed to the article and approved the submitted version.

## Conflict of Interest

XD, TZ, CD, PD, BK, SJ, and JY are stockholders of Predicine, Inc. PD, SJ, and JY are part of the leadership team of Predicine, Inc. FX, YZ, JD and YH are stockholders of Huidu Shanghai Medical Sciences, Ltd. MZ, RZ, and CL are stockholders of Laekna Therapeutics Shanghai Co., Ltd. YL and DW, and HL are stockholders of Shanghai Lide Biotech Co., Ltd.

The remaining authors declare that the research was conducted in the absence of any commercial or financial relationships that could be construed as a potential conflict of interest.

## Publisher’s Note

All claims expressed in this article are solely those of the authors and do not necessarily represent those of their affiliated organizations, or those of the publisher, the editors and the reviewers. Any product that may be evaluated in this article, or claim that may be made by its manufacturer, is not guaranteed or endorsed by the publisher.
