# Supplementary material for: Circulating Cell-Free DNA-Based Detection of Tumor Suppressor Gene Copy Number Loss and Its Clinical Implication in Metastatic Prostate Cancer
Source: Front Oncol. 2021 Aug 24;11:720727. doi: 10.3389/fonc.2021.720727 (PMC8422845; doi:10.3389/fonc.2021.720727)
Supplement: Supplementary file 1 [file DataSheet_1.docx]

**Supplementary Content**

| S Methods. | Blood collection and cfDNA extraction  Tissue processing for DNA extraction, targeted capture, sequencing, and bioinformatics  Analytical validation of CNV detection  Validation of copy number analysis using low-pass whole genome sequencing  Validation of copy number analysis using IHC |
| --- | --- |
|  |  |
| Table S1. | Assay performance metrics |
| Table S2. | Baseline patient characteristics |
| Table S3. | PredicineCARE cfDNA assay gene list |
| Table S4. | ctDNA fraction, NEPC status and detected PTEN/RB1/TP53 aberrations of 15 plasma samples in analytical validation cohort |
| Table S5. | Cell-free DNA sample information and sequencing metrics |
| Table S6. | Survival in mCRPC patients (before chemotherapy) based on ctDNA SNVs/CNVs gene events at the univariate and multivariate levels, adjusting for age, ctDNA fraction, cfDNA Yield, and Gleason Score |
|  |  |
| Figure S1. | Experimental workflow |
| Figure S2. | Cross genome segmented copy number estimation from matched blood and tissue samples of patient S10 using low-pass whole genome sequencing assay (LP-WGS) |
| Figure S3. | Correlation between tissue based and plasma cfDNA based copy number estimation using PredicineCARE assay |
|  |  |
|  |  |
|  |  |
|  |  |
|  |  |
|  |  |
|  |  |
|  |  |

# S Methods

## Blood collection and cfDNA extraction

Peripheral blood was collected, processed and cell-free DNA (cfDNA) isolated as previously described [1]. Briefly, a single tube (10 mL) of whole blood was collected in EDTA-containing tubes before undergoing two-step centrifugation to separate plasma and buffy coat. Aliquoted samples were stored at -80°C for batch processing. cfDNA was extracted from up to 5 mL of plasma using the QIAamp circulating nucleic acid kit (Qiagen, Hilden, Germany). Genomic DNA (gDNA) was extracted from buffy coat peripheral blood mononuclear cells (PBMCs) using the QIAamp DNA Blood Mini Kit (Qiagen), then enzymatically fragmented. Following quantification (Qubit 2.0 fluorometer, ThermoFisher Scientific, Waltham, Massachusetts, USA) and quality assessment (Bioanalyzer 2100, Agilent Technologies, California, USA) of extracted cfDNA and gDNA, large molecular weight DNA fragments were removed using AMPure XP beads (Beckman Coulter, California, USA). Five to 40ng of extracted cfDNA or fragmented gDNA was then utilized for library preparation.

**Tumour tissue collection and tDNA extraction**

Following metastatic tissue biopsy, one section of the biopsy underwent immediate fixation in 10% neutral formalin for 24 hours, followed by generation of formalin-fixed paraffin-embedded (FFPE) tissue blocks. The remaining section was first rinsed with Hanks’ balanced salt solution and sub-divided prior to digestion with collagenase solution for 1-2 hours at 37°C. The resulting single cells were then collected with subsequent depletion of immune and stromal cells using microbeads, enriching for tumour cells. Where insufficient tissue was available in the FFPE block for IHC (S4 and S5), these isolated tumour cells were used to generate FFPE blocks for PTEN IHC staining and tDNA extraction. Among all tissue samples, nine tissue samples (S2, S4, S7, S8, S9, S10, S11, S12, S14) were reviewed as neuroendocrine-like prostate cancer based on their characteristics of histopathology and nuclear morphology as described in the Supplementary Table S4.

**Tissue-based PTEN immunohistochemistry**

PTEN protein expression was determined by IHC on 3 µM FFPE sections using the UltraVision Quanto Detection System HRP DAB (Thermo Fisher Scientific, Waltham, Massachusetts, USA) and the138G6 rabbit monoclonal antibody (Cell Signaling Technology, Danvers, MA, USA). Following deparaffinization, antigen retrieval was conducted in sodium citrate buffer at 100°C for 20 min. The slides were then washed with phosphate buffered saline (PBS) and incubated in UltraVision Hydrogen Peroxide Block for 10 min. After further PBS washings, slides were incubated for 10 min with the UltraVision Protein Blocking solution. The slides were then incubated with the primary antibody overnight at 4°C at a dilution of 1:400. The following day, slides were washed four times with PBS and incubated with Primary Antibody Amplifier Quanto for 10 min. Another buffer wash step was then performed, before applying HRP Polymer Quanto and incubating for 10 min. DAB Quanto Chromogen (30 µl) was mixed with DAB Quanto substrate (1 ml), applied to the slides and incubated for five minutes. The slides were then washed with water and counterstained with hematoxylin.

Slides were subjected to blinded review by two independent pathologists. First, both PTEN staining intensity and staining area were individually scored from 0-3, with the two values multiplied to produce an overall staining score (0-9). The final PTEN IHC score was evaluated as follows: staining score of 0-1 was referred to as absent staining (–), 2-3 as weak staining (1+), 4-6 as moderate staining (2+), and 7-9 as strong staining (3+). Patient-derived xenograft tissues with low and high PTEN expression were used as positive and negative controls for IHC staining.

## Targeted capture, sequencing and bioinformatics

### Library preparation, hybrid-capture and sequencing

Preparation of next-generation sequencing libraries began with DNA end-repair, dA-tailing, adapter ligation and PCR amplification as previously outlined [1]. Amplified DNA libraries subsequently underwent further quality control (Bioanalyzer 2100), before being hybridized overnight to a custom-designed targeted panel capturing exonic regions from 90 genes (**see below**). Captured fragments were recovered, washed and further PCR amplified. A final quality control assessment (Bioanalyzer 2100) was performed to confirm the presence of a dominant peak at approximately 300 bp and adequate library quantity (fragments between 200-600 bp >1 nM). Enriched libraries were then sequenced on the Illumina HiSeq X Ten. Genes included in the Predicine cfDNA assay are shown in **Table S3** of the **Supplement**.

### Sequence alignment and quality control

Paired-end reads underwent quality control and sequence alignment using an in-house pipeline that performs barcode checking, adapter trimming, and error correction. Cleaned paired FASTQ files were aligned to the hg19 reference genome using the BWA alignment tool [2].

### Somatic mutation identification

Consensus BAM files were derived as previously described to minimize sequencing and PCR errors [1]. An in-house pipeline was used to identify candidate variants whilst filtering/removing low-quality variants. A variant was considered a candidate somatic mutation only when all four of the following conditions were met: i) at least four distinct fragments contained the mutation, ii) variant allelic frequency (AF) was at least 0.25%, or 0.1% for hotspot loci (as defined by COSMIC and <http://www.cancerhotspots.org>), iii) variant was absent from public databases of common germline variants (1000 genomes, ExAC, gnomAD and KAVIAR), and iv) variant was not present in matched PBMC samples with variant frequency >5%. This threshold was chosen on the basis that the mean unique read depth for our germline DNA samples was 100-200x. This depth allowed us to reliably detect variants down to 5% mean allele frequency (MAF) when at least 8 supporting reads were required. Candidate somatic mutations were further annotated and filtered to include only missense, nonsense, frameshift, or splice site variants occurring in protein-coding regions. Predicted benign variants (ClinVar) and previously described haematopoietic expansion-related variants [3] were also removed.

### Germline DNA analysis

Germline variants were determined by concurrent sequencing of buffy coat PBMCs. Candidate variants with low base quality, mapping scores, and other quality metrics were removed. Candidate variants with an allelic frequency <5%, or with less than eight distinct reads containing the mutation, were excluded. Unknown variants in repeat regions were also excluded.

### Copy number analysis by targeted panel

Estimation of panel-based copy number variation occurred at the gene level. In-house algorithms calculated the on-target unique fragment coverage based on the consensus BAM file, followed by GC bias correction. Each adjusted coverage profile was self-normalized and then compared against correspondingly adjusted coverages from a group of normal reference samples to estimate the significance of the copy number variation. The minimum gain or loss thresholds were determined based on the CNV change distribution of normal reference samples. Gains or deletions with an absolute z-score > 3 (the inverse of the cumulative distribution function of a Gaussian distribution at 99.59% confidence level) and absolute CNV change above minimum gain or loss thresholds were called as true events.

To increase the specificity of copy number calls, the pipeline integrates the variant allele frequency information of common SNPs located up to 1 Mb upstream and downstream of the genes in panel. If there is only one SNP allele with altered MAF or with a significantly different copy number to the other allele, then the allele variant frequency of the heterozygous SNPs will shift away from the expected 0.5. We define the average variant frequency deviation from 0.5, ${AF}_{shift}$, as


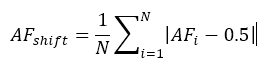


where ${AF}_{i}$ is the variant frequency of the ${SNP}_{i}$, N is the number of heterozygous SNPs located in the gene region (including up to 1Mb upstream and downstream of the gene body). The pipeline considers the change of ${AF}_{shift}$ is significant if


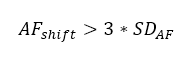


where ${SD}_{AF}$ is the standard deviation of SNP variant frequency ${AF}_{i}$, and at least 3 supporting heterozygous SNPs ($N\geq3$) are required to call a significant ${AF}_{shift}$. The CNV pipeline detects a gene with CNV changes if it satisfies both copy number changes and ${AF}_{shift}$ thresholds. For genes without heterozygous SNP support, or having heterozygous SNP coverage but lacking of SNP ${AF}_{shift}$ support, a more stringent gene copy number change threshold (1.5x of minimum copy number change threshold) is applied to make a confident CNV call.

## Validation of copy number analysis using low-pass whole genome sequencing

Low-pass whole genome sequencing (LP-WGS) with an overall average coverage of 2x was performed on the 15 tissue samples used in clinical validation. The ichorCNA tool algorithm [3] with default parameter settings (1Mb partitioning) was applied to GC and mappability-normalized reads to estimate plasma copy number variations using a hidden Markov model.

# References

[1] Heidi Fettke, Edmond M. Kwan, *et. al.* Combined cell-free DNA and RNA profiling of the androgen receptor: Clinical utility of a novel multianalyte liquid biopsy assay for metastatic prostate cancer. *European Urology*, 78(2):173–180, aug 2020.

[2] H. Li and R. Durbin. Fast and accurate short read alignment with burrows-wheeler transform. *Bioinformatics*, 25(14):1754–1760, may 2009.

[3] Viktor A. Adalsteinsson, Gavin Ha, *et. al*. Scalable whole-exome sequencing of cell-free DNA reveals high concordance with metastatic tumors. *Nature Communications*, 8(1), nov 2017.

**Supplementary Figures:**

**
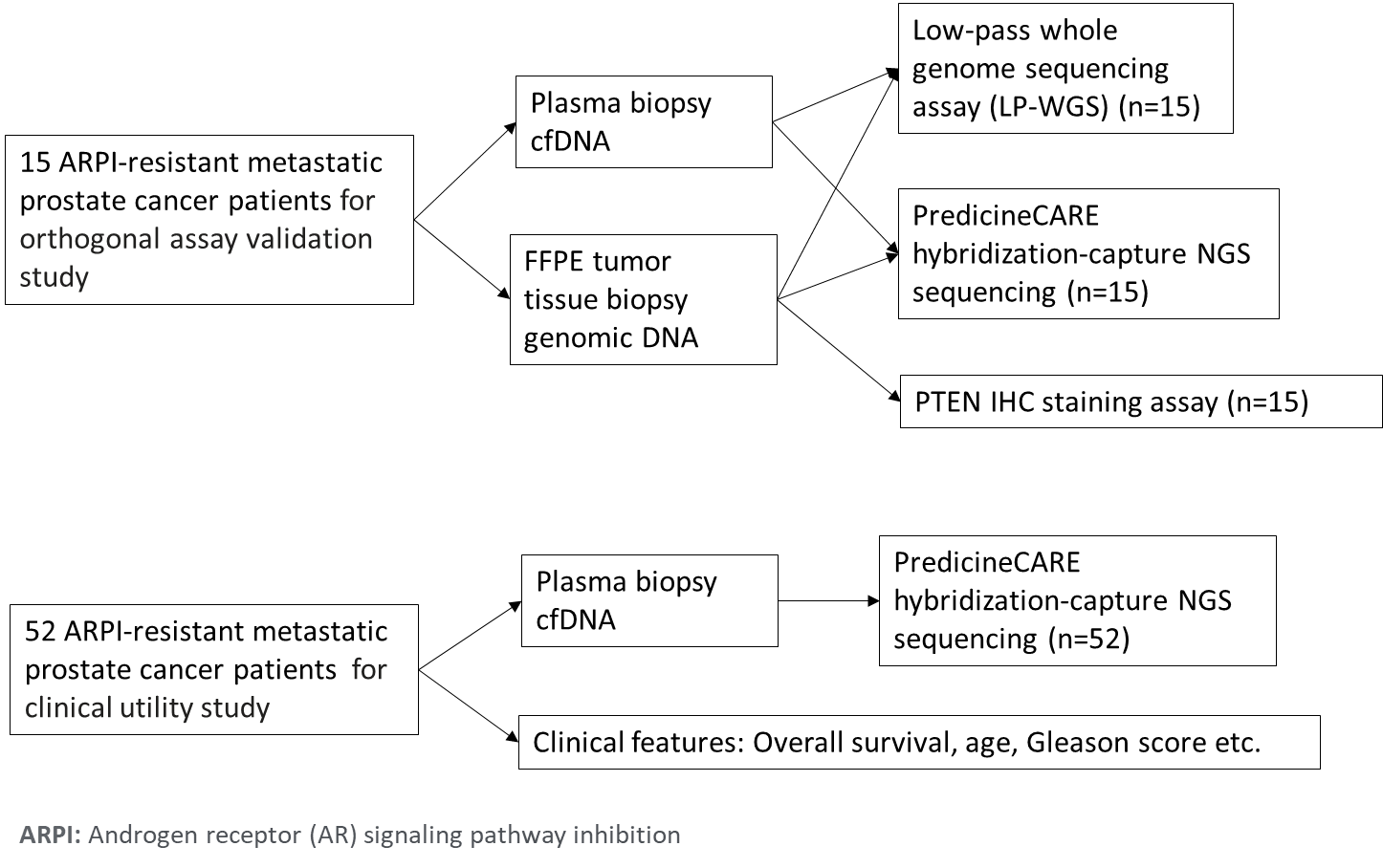
**

**Figure S1. Experimental workflow**


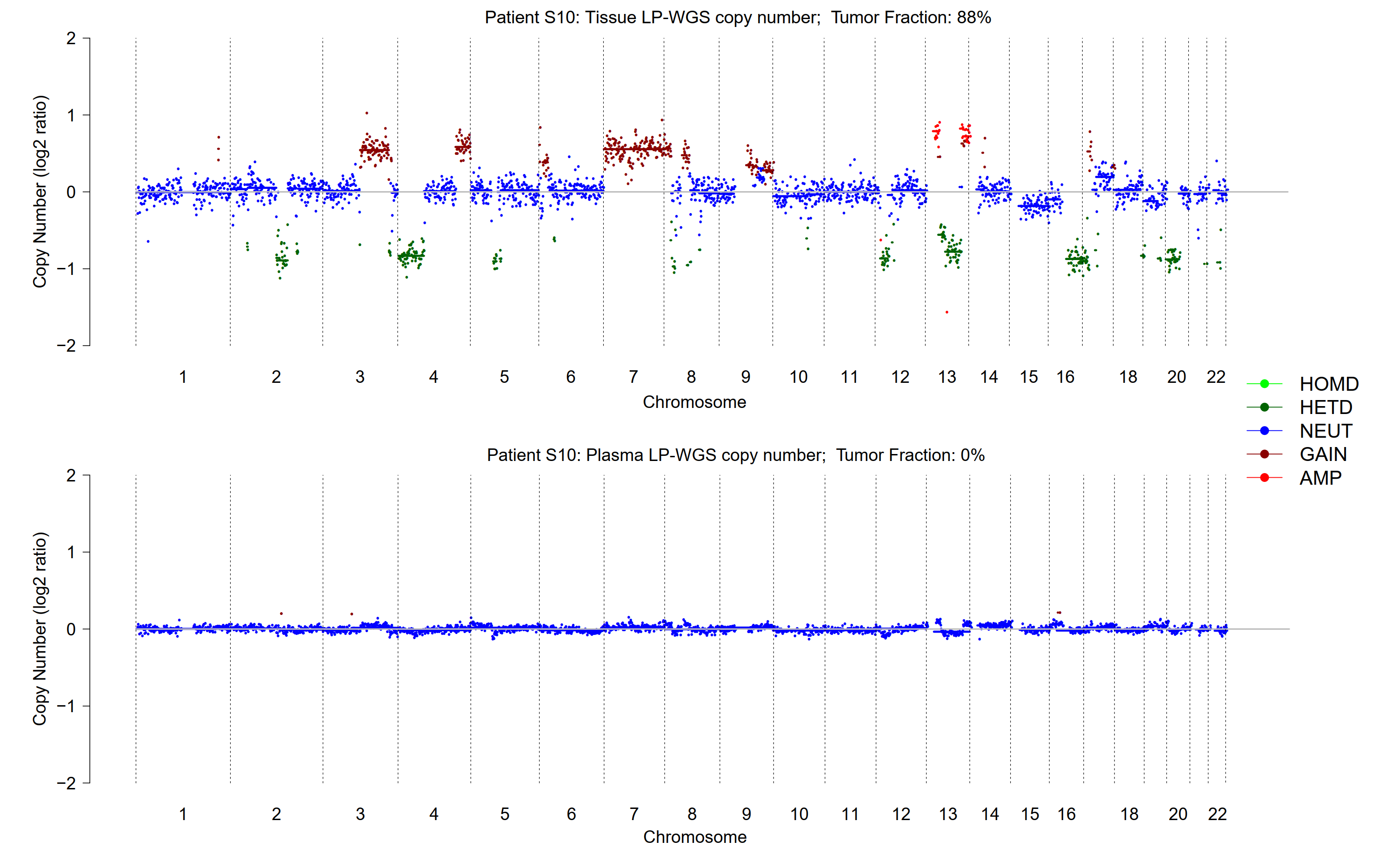


**Figure S2.** Cross genome segmented copy number estimation from matched blood and tissue samples of patient S10 using low-pass whole genome sequencing assay (LP-WGS). Each data point represents the copy number estimation for a 1 million base pair region. For each region, copy number status was estimated using ichorCNA. HOMD: homozygous deletion; HETD: heterozygous deletion; NEUT: neutral copy number; GAIN: copy gain, AMP: amplification.

**
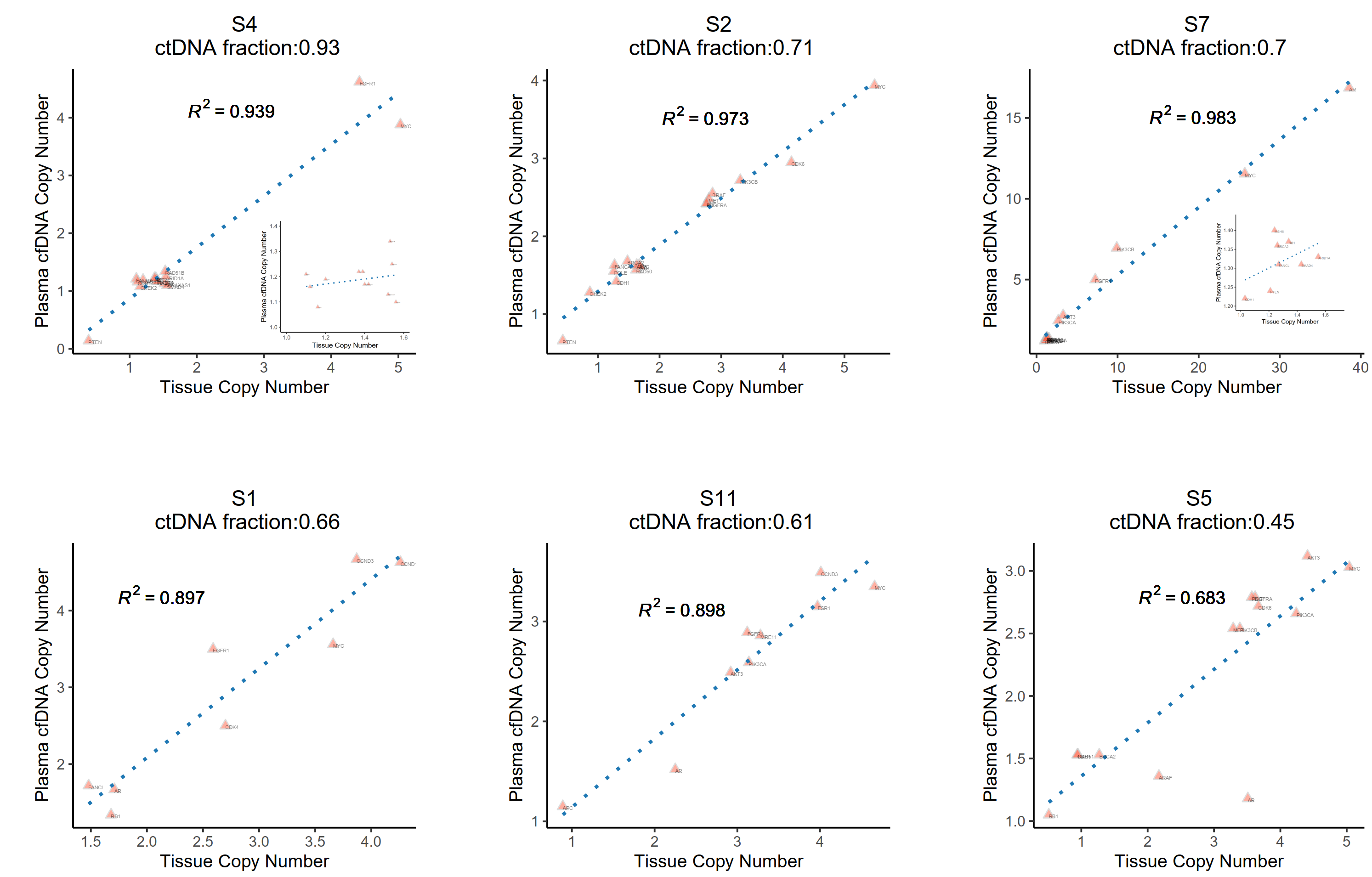
**

## Figure S3. Correlation between copy numbers estimated from liquid and tissue biopsies for genes with both tissue and liquid biopsy CNV calls in the 6 pairs of samples that have >4 copy number events. Each gene is represented as a single data point. Blue dashed line represents the fitted linear regression line.

**Supplementary Tables:**

**Table S1: Performance metrics of PredicineAssay**

|  | **Assay Performance** |
| --- | --- |
| **Accuracy** | 99.7% [98.5-100%] |
| **Precision - Intra** | 97.8% [86.5-99.9] |
| **Precision - Inter** | 99.6% [97.5-100] |
| **Limit of Detection** | \|  \| LOD \| Sensitivity \| \| --- \| --- \| --- \| \| SNV \| 0.25% MAF \| 95.8% [90.5-98.6%] \| \| Indel \| 0.25% MAF \| 95.7% [88-99.1%] \| \| Fusion \| 0.375% MAF \| 96.7% [82.8-99.9%] \| \| CNG \| 2.23 - 2.37 Copies \| 100% [69.2-100%] \| \| CNL \| 1.75 copies \| 100% [83.9-100%] \| \|  \|  \|  \| |
| **Analytical specificity** | 99.99995 % |

**Table S2. Baseline patient characteristics**

**A. Orthogonal validation cohort: 15 patients.**

|  |  |
| --- | --- |
| **Characteristics** | No. (%) |
| **Age in years at the time specimen collection, median (range)** | 65 (42 - 80) |
| **Clinical TNM staging at ID, no.** |  |
| T1 | 0 (0) |
| T2 | 0 (0) |
| T3 | 1 (6.7) |
| T4 | 2 (13.3) |
| T-unknown | 13 (86.7) |
| N0 | 0 (0) |
| N1 | 8 (53.3) |
| N-unknown | 7 (46.7) |
| M0 | 0 |
| M1 | 15 (100) |
| M-unknown | 0 |
| **Bone metastasis** | 15 (100) |
| **Lung metastasis** | 3 (20) |
| **Liver metastasis** | 2 (13.3) |
| **Other metastasis** | 8 (53.3) |
| **Prior abiraterone** | 15 (100) |
| **Prior enzalutamide** | 1 (6.7) |
| **Prior abiraterone + enzalutamide** | 1 (6.7) |

**B. Clinical utility study cohort: 52 patients.**

|  | **Before Chemotherapy (n = 52)** |
| --- | --- |
| **Patients with analyzable NGS data (N)** | 52 |
| **Age in years at the time specimen collection, median (range)** | 72 (50 - 87) |
| **Gleason Score at ID, no.** |  |
| ≤ 7 | 20 |
| ≥ 8 | 28 |
| Unknown | 4 |
| **Clinical TNM staging at ID, no.** |  |
| T1 | 2 |
| T2 | 16 |
| T3 | 22 |
| T4 | 1 |
| T-unknown | 11 |
| N0 | 20 |
| N1 | 11 |
| N-unknown | 21 |
| M0 | 38 |
| M1 | 12 |
| M-unknown | 2 |
| **PSA at time of sample collection, median ng/ml (IQR)** | 18.3 (6.7 - 76.2) |
| **ALP at the time of sample collection, median (range)** | 106.5 (39 - 2185) |
| **LDH at the time of sample collection, median (range)** | 213 (137 - 384) |
| Patients with missing values, no. | 33 |
| **Hemoglobin at sample collection, median (range)** | 12.7 (8.4 - 14.8) |
| Patients with missing values, no. | 17 |
| **Radical prostatectomy on ID, no.** | 26 |
| **Radiation alone on ID, no.** | 6 |
| **Radical prostatectomy and radiation on ID, no** | 0 |
| **Salvage local treatments after primary prostate treatments** | 19 |
| **Median time from initial treatments for localized stage disease to disease progression, mo (range)** | 19.8 (3.8 - 203.2) |
| **Received docetaxel after clinical progression, no.** | 52 |
| **Median time from ADT initiation for mHSPC stage to biochemically progress to CRPC stage, mo (range)** | 12.2 (3.2 - 203.0) |
| **Median follow-up time from date of mCRPC specimen collection to last follow up, mo (range)** | 98.7 (72.1 - 110.6) |
| **Median time to death/last follow-up for mCRPC patients, mo (range)** | 23.9 (3.0 - 94.7) |
| **Patients dead upon follow-up, no.** | 51 |

**Table S3. PredicineCARE cfDNA assay gene list**


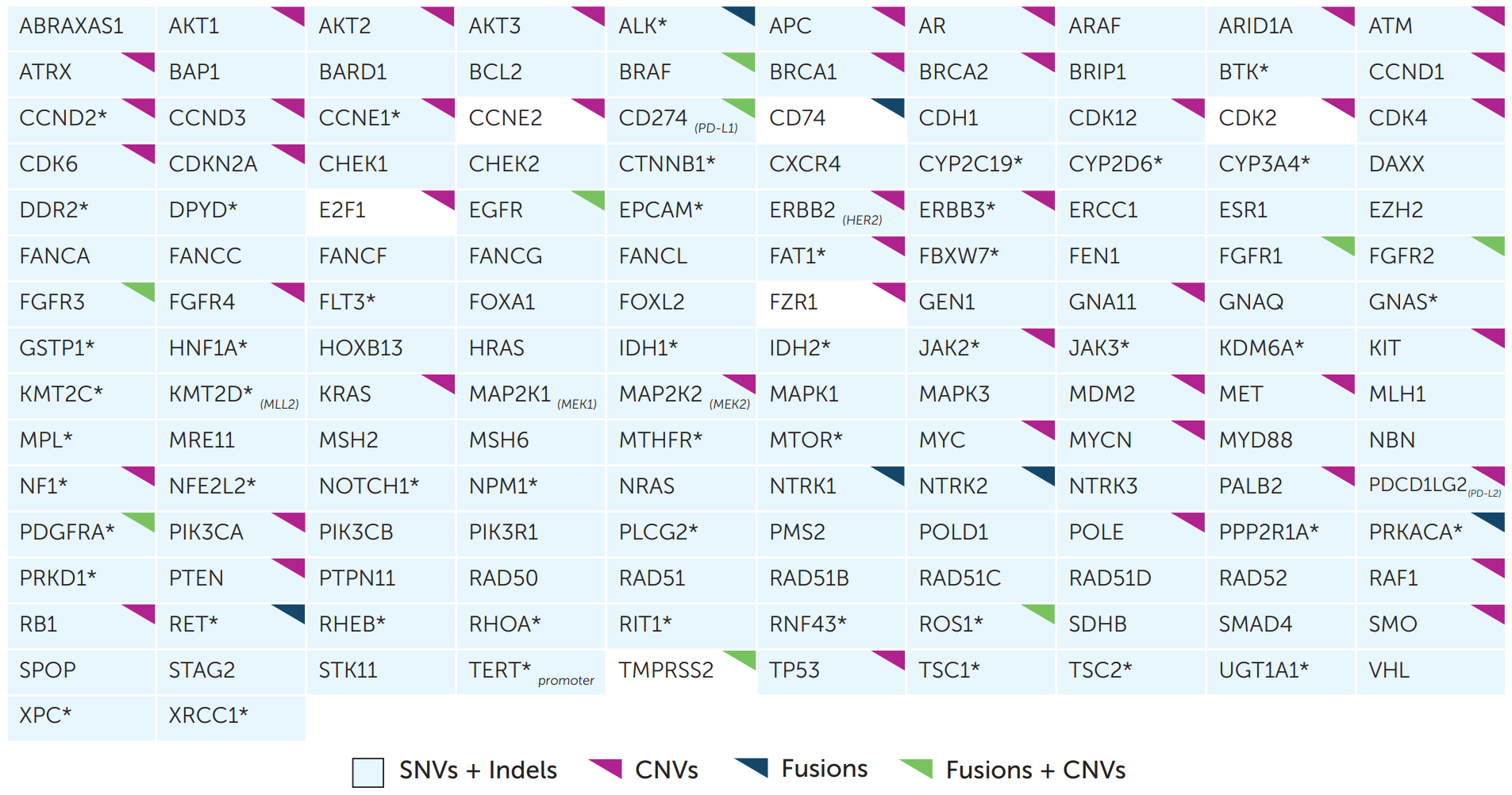


**Table S4. Tumor content, NEPC status and detected PTEN/RB1/TP53 aberrations of 15 plasma samples in the analytical validation cohort.**

| **Manuscript SampleID** | **Tumor Content by H&E** | **Pathology Reviewer 1** | **Pathology Reviewer 2** | **NEPC** | **TP53** | **RB1** | **PTEN** | **Tumor Fraction (Plasma)** | | **Tumor Fraction (FFPE)** | |
| --- | --- | --- | --- | --- | --- | --- | --- | --- | --- | --- | --- |
|  |  |  |  |  |  |  |  | LP-WGS | PredicineCARE | LP-WGS | PredicineCARE |
| S1 | <1% | ++ | ++ |  |  | CNL |  | 0.524 | 0.425 | 0.098 | 0.296 |
| S2 | >20% | - | - | Likely | c.844C>T | CNL | CNL | 0.402 | 0.714 | 0.515 | 0.86 |
| S3 | 10-20% | - | - |  | c.920-1G>A |  | CNL | 0.126 | 0.326 | 0.256 | 0.528 |
| S4 | ~20% | - | - | Likely | c.707A>G | c.234_250del;CNL | CNL | 0.871 | 0.963 | 0.878 | 0.938 |
| S5 | ~20% | ++ | ++ |  |  | CNL |  | 0.403 | 0.181 | 0.809 | 0.544 |
| S6 | 5% | ++ | ++ |  |  |  |  | 0 | 0.013 | 0.026 | 0.006 |
| S7 | >20% | - | - | Likely | c.733G>A | CNL | CNL | 0.644 | 0.69 | 0.887 | 0.911 |
| S8 | >20% | - | + | Likely | c.848G>C | CNL | CNL | 0.152 | 0.206 | 0.533 | 0.754 |
| S9 | >20% | - | - | Likely |  | CNL | CNL | 0.727 | 0.774 | 0.311 | 0.655 |
| S10 | >20% | - | - | Likely | c.844C>T | CNL | c.362C>A;CNL | 0 | 0.085 | 0.884 | 0.92 |
| S11 | >20% | - | - | Likely |  |  | c.43A>G;CNL | 0.496 | 0.609 | 0.796 | 0.885 |
| S12 | >20% | - | - | Likely | c.714T>G | CNL | c.144C>A;CNL | 0 | 0.013 | 0.669 | 0.684 |
| S13 | 10-20% | + | - |  |  | CNL | CNL | 0.251 | 0.114 | 0.026 | 0.006 |
| S14 | >20% | ++ | ++ | Likely | c.902del |  |  | 0.04 | 0.149 | 0.257 | 0.879 |
| S15 | >20% | ++ | ++ |  |  |  |  | 0 | 0.006 | 0.829 | 0.216 |

**Table S5. Cell-free DNA sample information and sequencing metrics. Please refer to separate data file in MS Excel.**

**Table S6. Association of PTEN/RB1 copy number loss with overall survival in 52 mCRPC patients by the univariate and multivariate Cox-PH models. The prognostic effect of individual gene was adjusted by age, ctDNA fraction, cfDNA Yield, and gleason score in the multivariate models.**

| **Gene** | **Aberration** | **Patients with mutation, no. (%)** | **Median OS, mo** | **Univariate** | |  | **Multivariate** | |
| --- | --- | --- | --- | --- | --- | --- | --- | --- |
|  |  |  |  | **HR (95% CI)** | ***P* value** |  | **HR (95% CI)** | ***P* value** |
| PTEN | CNV | 10 (19.2) | 14.1 | 3.2 (1.5-6.8) | 0.002 |  | 2.8 (1.2-6.9) | 0.02 |
| RB1 | CNV | 15 (28.8) | 13.3 | 4.5 (2.3-8.8) | 2.2E-06 |  | 6.7 (2.8-16.2) | 2.2E-05 |

**HR and *P* value of each feature in multivariate models:**

| **Gene** | **Features** | **HR (95% CI)** | ***P* value** |
| --- | --- | --- | --- |
| PTEN | PTEN detected | 2.8 (1.2-6.9) | 0.02 |
|  | cfDNA yield | 1.3 (0.6-2.7) | 0.48 |
|  | ctDNA fraction | 2.0 (0.9-4.6) | 0.09 |
|  | Gleason score | 1.7 (0.8-3.3) | 0.16 |
|  | age | 1.0 (0.97-1.1) | 0.37 |
| RB1 | RB1 detected | 6.7 (2.8-16.2) | 2.20E-05 |
|  | cfDNA yield | 1.3 (0.7-2.5) | 0.41 |
|  | ctDNA fraction | 1.9 (0.8-4.3) | 0.13 |
|  | Gleason score | 1.6 (0.8-3.1) | 0.19 |
|  | age | 1.0 (0.98 - 1.07) | 0.31 |
